# Neurobiological Mechanisms of Metacognitive Therapy – An Experimental Paradigm

**DOI:** 10.3389/fpsyg.2019.00660

**Published:** 2019-04-04

**Authors:** Lotta Winter, Mesbah Alam, Hans E. Heissler, Assel Saryyeva, Denny Milakara, Xingxing Jin, Ivo Heitland, Kerstin Schwabe, Joachim K. Krauss, Kai G. Kahl

**Affiliations:** ^1^Department of Psychiatry, Social Psychiatry and Psychotherapy, Hannover Medical School, Hanover, Germany; ^2^Department of Neurosurgery, Hannover Medical School, Hanover, Germany; ^3^Center for Stroke Research Berlin, Charité – Berlin University of Medicine, Berlin, Germany; ^4^Department of Neurosurgery, Zhongda Hospital, Southeast University, Nanjing, China

**Keywords:** metacognitive therapy, local field potential, deep brain stimulation, treatment resistance, BNST/IC

## Abstract

**Introduction:**

The neurobiological mechanisms underlying the clinical effects of psychotherapy are scarcely understood. In particular, the modifying effects of psychotherapy on neuronal activity are largely unknown. We here present data from an innovative experimental paradigm using the example of a patient with treatment resistant obsessive-compulsive disorder (trOCD) who underwent implantation of bilateral electrodes for deep brain stimulation (DBS). The aim of the paradigm was to examine the short term effect of metacognitive therapy (MCT) on neuronal local field potentials (LFP) before and after 5 MCT sessions.

**Methods:**

DBS electrodes were implanted bilaterally with stereotactic guidance in the bed nucleus of the stria terminalis/ internal capsule (BNST/IC). The period between implantation of the electrodes and the pacemaker was used for the experimental paradigm. DBS electrodes were externalized via extension cables, yielding the opportunity to record LFP directly from the BNST/IC. The experimental paradigm was designed as follows: (a) baseline recording of LFP from the BNST/IC, (b) application of 5 MCT sessions over 3 days, (c) post-MCT recording from the BNST/IC. The Obsessive-Compulsive Disorder- scale (OCD-S) was used to evaluate OCD symptoms.

**Results:**

OCD symptoms decreased after MCT. These reductions were accompanied by a decrease of the relative power of theta band activity, while alpha, beta, and gamma band activity was significantly increased after MCT. Further, analysis of BNST/IC LFP and frontal cortex EEG coherence showed that MCT decreased theta frequency band synchronization.

**Discussion:**

Implantation of DBS electrodes for treating psychiatric disorders offers the opportunity to gather data from neuronal circuits, and to compare effects of therapeutic interventions. Here, we demonstrate direct effects of MCT on neuronal oscillatory behavior, which may give possible cues for the neurobiological changes associated with psychotherapy.

## Introduction

With the introduction of neuroimaging techniques in psychotherapy research, the neurobiological correlates of psychotherapeutic interventions have been increasingly investigated (Yang et al., 2014). A number of studies suggest that the progress and outcome of psychotherapy can be associated with neurobiological changes (Messina et al., 2013; Barsaglini et al., 2014; Weingarten and Strauman, 2015). Neuroimaging studies about psychotherapy effects have, however, only roughly demonstrated that changes in cognition and behavior through psychotherapy (mainly cognitive behavioral therapy; CBT) and neuronal changes in the brain are somehow interrelated. Accordingly, the exact moderating and mediating effects of psychotherapy on neuronal substrates are largely unknown (Sakai et al., 2006; Yang et al., 2014).

Obsessive-compulsive disorder (OCD) is a severe psychiatric illness, which is treated by psychotherapy and pharmacotherapy in the first instance according to current guidelines (National Institute for Health and Care Excellence [NICE], 2005; Deutsche Gesellschaft fuer Psychiatrie und Psychotherapie, Psychosomatik und Nervenheilkund e.V [DGPPN], 2013), with response and remission rates between 20 and 70% depending on the kind of treatment and the measured criterion (Fisher and Wells, 2005; Grados and Riddle, 2008). Metacognitive therapy (MCT) is a modern development in psychotherapy standing out by comparably short treatment duration, high effect sizes and transdiagnostic effects considering comorbid disorders (Normann et al., 2014; Sadeghi et al., 2015; van der Heiden et al., 2016). MCT is a cognitive therapy derived from the Metacognitive theory of psychological disorders (Wells and Matthews, 1994). Referring to its distinctive theoretical origin MCT focusses on metacognitive processes and metacognitive beliefs as well as on regulating thinking styles. This is in contrast to traditional cognitive therapy where cognitive content is the target of psychotherapeutic intervention. For people suffering from OCD it is a promising treatment option (van der Heiden et al., 2016). The effects of MCT on neurophysiological mechanisms which lead to clinical improvement have not been elucidated so far.

Brain function in OCD has been investigated using functional magnetic resonance imaging, structural brain morphology, positron emission tomography and EEG methods (Linden, 2006; O’Neill et al., 2013; Dohrmann et al., 2017; Moody et al., 2017; Atmaca et al., 2018). In particular, hyperactivity of the cortico-striato-thalamo-cortical (CSTC) circuit has been proposed as the neurobiological basis of OCD (Saxena et al., 2001). This concept achieved further support by studies demonstrating increased cerebral blood flow in the CSTC by symptom provocation (McGuire et al., 1994; Rauch et al., 1994; Adler et al., 2000), and decreased activation after treatment with selective serotonin-reuptake inhibitors or psychotherapy (Brody et al., 1998).

However, the CSTC model does not take into consideration the role of the amygdala and its interaction with the frontal lobe in mediating fear and anxiety in OCD (Milad and Rauch, 2012). The amygdala and the associated bed nucleus of the stria terminalis (BNST, also called the extended amygdala) constitute an integrative center for emotions and emotional behavior, whose role in mediating fear and anxiety in OCD is a hotspot of current research (Lesting et al., 2011; Daldrup et al., 2016; Kohl et al., 2016).

Deep brain stimulation (DBS) targeting the BNST and the neighboring internal capsule (IC) is a novel therapeutic strategy in treatment resistant OCD (trOCD) that exerts its effects via electric stimulation, thereby possibly modulating the activity of pathological neuronal circuits (Naesstrom et al., 2016). In line with this, imaging and DBS studies suggest that the BNST and orbital frontal cortex are implicated in the pathophysiology of OCD (Luyten et al., 2016).

The DBS treatment approach provides a unique opportunity to study the neural activity of subcortical brain areas in patients. Further, postoperative recording via externalized leads of the electrodes provides the opportunity to gather data on brain activity in pathological disease states as well as changes of brain activity after psychotherapeutic intervention. We here present a new experimental paradigm to investigate the neuronal effects of psychotherapy, exemplified with MCT, in a patient with trOCD treated with DBS.

## Materials and Methods

### Operative Procedure

The data reported in this study were recorded from a 51-year-old left-handed male with drug- and CBT- refractory OCD, who underwent implantation of DBS electrodes in the bed nucleus of the stria terminalis/ internal capsule (BNST/IC) bilaterally. This patient showed OCD symptoms mainly in the domains of checking, ordering and symmetry with an onset in the 1980s. In the pre-assessment prior to the first surgery he presented a sum score of 39 on the German version of the Yale Brown Obsessive Compulsive Scale (Y-Bocs), (Hand and Büttner-Westphal, 1991; Jacobsen et al., 2003). A current depressive episode could be excluded. The patient was drug free during the study procedure. Before DBS the patient was treated according to the German S3-guidelines for OCD (Deutsche Gesellschaft fuer Psychiatrie und Psychotherapie, Psychosomatik und Nervenheilkund e.V [DGPPN], 2013), and had received two qualified treatments using disorder specific cognitive-behavioral therapy including exposure and response prevention, combined with recommended drug treatments. Currently MCT is not part of this guideline, and was therefore not considered before DBS treatment. The study was approved by the Ethics Committee of Hannover Medical School and the patient gave written informed consent prior to the study onset.

The quadripolar DBS electrodes (model 3387, Medtronic, Minneapolis, MN, United States) had four platinum-iridium cylindrical contact surfaces (1.27 mm diameter and 1.5 mm length) and a contact-to-contact separation of 1.5 mm. DBS electrodes were implanted bilaterally with CT-stereotactic guidance, aided by magnetic resonance imaging, and microelectrode recording in the BNST/IC under local anesthesia. Microelectrode recording was used to define the trajectory within BNST and IC. Contact 0, the lowermost contact, was placed in the BNST, and the upper contacts were placed in the IC. Details of target localization during the intraoperative procedure and implantation of the neurostimulation system are described elsewhere (Winter et al., 2018). Appropriate electrode placement was confirmed by postoperative stereotactic CT. The implantable pulse generator was implanted under general anesthesia. Appropriate electrode placement was confirmed by postoperative stereotactic CT.

### Experimental Design

The paradigm was part of a larger study on the effects of DBS of the BNST/IC in OCD (in preparation). The time period between implantation of DBS electrodes and the implantable pulse generator (IPG) was used for the experiments. During this period LFPs were obtained directly from the contacts in the BNST/IC. Elements of MCT were applied five times and neurophysiological oscillatory activity was recorded via the DBS electrodes before and after MCT. No stimulation was performed during this period. Table 1 presents an overview of the protocol.

**Table 1:** Study protocol.

Day	Procedure	Time
One week before	Pre-assesment (Y-Bocs, HamD, BDI, OCD-S),	T0
Day 1	Surgery: implantation of electrodes	
Day 2	One day break	
Day 3	Neurophysiological recordings MCT 1 Half day break MCT 2 OCD-S	Pre-therapy T1
Day 4	MCT 3 Half day break MCT 4 OCD-S	T2
Day 5	one day break (to practice ATT)	
Day 6	MCT 5 Neurophysiological recordings Half day break OCD-S	Post-therapy T3
Day 7	Surgery: implantation of IPG	

*Y-Bocs, Yale Brown Obsessive Compulsive Scale; HamD, Hamilton Depression Scale; BDI-II, Beck Depression Inventory II; OCD-S, Obsessive-Compulsive Disorder Scale; MCT, metacognitive therapy; ATT, Attention Training Technique; IPG, implantable pulse generator.*

### Metacognitive Therapy

Metacognitive therapy is a theory-based development in modern psychotherapy. Founded on the Self-Regulatory Executive Function Model (S-REF) (Wells and Matthews, 1994), MCT postulates that psychiatric disorders are a result of disturbed information processing. Perseverative thinking styles and inflexible attention patterns are maintained by unhelpful metacognitions. The aim of the treatment is to help the patient develop new ways of controlling attention, relating to thoughts and inner events and modify underlying metacognitions. Part of the intervention strategies is to practice detached mindfulness and attention training. With detached mindfulness the patients can develop the experience that one can step back from thoughts and other inner events and let these control themselves without doing anything actively. This experience can be presented and practiced using different metaphors and exercises described in the treatment manual (Wells, 2009). Attention training (ATT) aims to help strengthen the awareness of attentional control (Wells, 1990). To practice, a sound file can be used. The training consists of actively listening to several presented sounds. Instructions help to focus and regulate attention in three phases. The first phase is to practice selective attention. Here the task is to focus on individual sounds whilst trying not to get distracted by other sounds. The second phase involves rapid switching of attention between different sounds and spatial locations. The last phase practices dividing attention by trying to widen the attention to attend as many sounds as possible.

According to the manual average treatment duration is around 12 sessions. As the time between implantation of the electrodes and the stimulator is limited the paradigm gives time for five sessions only. Therefore the content of the sessions does not follow the manual. We chose to investigate two components of therapy which are detached mindfulness and ATT. They were both practiced with the patient. In the first session, he was provided with an individual case formulation of his OCD to socialize the concept. In this session, detached mindfulness was introduced. Detached mindfulness was also trained in session 2. In session 3, ATT was introduced and the audio file of the German version of ATT was provided for practice. The patient was asked to practice at least three times per day on that day and the next day. ATT and practice of detached mindfulness were repeated in session 4. Session 5 consisted of supervised ATT only. Each session lasted approximately 45 min.

### Psychometric Measures

The German version of the Obsessive-Compulsive Disorder Scale (OCD-S) (Wells, 2009) was used to evaluate the effect of MCT subjectively. The focus was to get information on the effects MCT may have even in this treatment resistant case. Instead of following the original instruction to rate the items considering the last week the patient was asked to refer to the time frame since he last answered the questionnaire. The OCD-S is a self-rating-scale used in MCT to evaluate the therapy progress. The questionnaire consists of four main questions and 22 sub-items. The patient was asked to rate items 1 to 3 on a scale ranging from 0 to 8. Item 4 asks for percentages (0–100%). As shown in Table 1 the OCD-S was obtained before surgery (T0), after session 1 (T1), after session 3 (T2) and after the last session (T3).

### Local Field Potentials and EEG Recording

**FIGURE 1:**
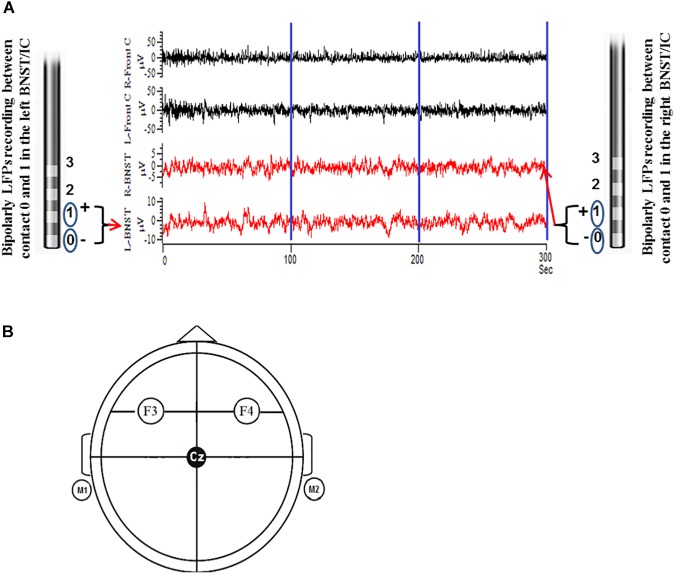
Illustration shows the LFPs were recorded as bipolar configuration from DBS electrodes from contacts 0 and 1 before and after MCT for a total of 300 s (100 s epoch was preferentially used to fulfill the stationarity criteria) **(A)**. Parallel EEG was recorded as unipolar configuration according to 10/20 system from cortical surface electrodes F3 and F4 (frontal cortical/motor planning area). F3 and F4 electrodes were linked together with left and right mastoid (M1 + M2) as reference **(B)**.

The electrophysiological recordings were undertaken 2 days after the implantation of the electrodes prior to the first MCT session and on day 6 after the last MCT session (see Table 1). DBS leads were still externalized during this time frame. The EEG and LFP recording was made in a resting condition. The patient sat in an arm chair in a relaxed and calm condition. We explicitly instructed the patient not to move the head or body and to keep his eyes open. The recording was running for at least 300 s (Figure 1A,B).

The local field potentials (LFP) were obtained from adjacent bipolar contact pairs (0 to 1) in the left and right BNST from the implanted DBS electrodes. LFP signals were amplified 50.000 fold and filtered (bandwidth 0.5–100 Hz) using a D360 amplifier (Digitimer Ltd., Welwyn Garden City, Hertfordshire, United Kingdom) at a sampling rate of 512 Hz through a 1401 A-D converter (CED, Cambridge, United Kingdom) onto a computer using Spike2 software. Simultaneous surface EEG recordings were taken over frontal cortical areas (F3 and F4) according to the International 10–20 System using Ag–AgCl contact surface electrodes referenced to the mastoid and band pass filtered at (0.5–100 Hz) and the sampling rate was 512 Hz. Electrode impedances were kept below 2 kΩ.

### Local Field Potentials and EEG Data Analysis

Due to an expected intrinsic non-stationarity in the LFP and EEG signals we segmented 300 s recorded data in to three epochs of equal length (3 × 100 s) for power of spectral analysis in different frequency bands e.g., theta, alpha, beta, and gamma. The analysis of spectral power or coherence of neural oscillatory activity measured in EEG and LFPs have provided a new insight into brain mechanisms of information processing in different neurological and neuropsychiatric disorders (Marceglia et al., 2007; Uhlhaas and Singer, 2010; Bowyer, 2016).

Three epochs of 100 s without major artifacts were used for frequency-domain signal processing from simultaneous recordings of BNST/IC LFP and frontal cortical EEG. After eliminating 50 Hz artifacts using a finite impulse response (FIR) notch filter, data were normalized by subtracting the mean amplitude and dividing the standard deviation, which allowed the frequency domain signals to be pooled and compared with less influences from individual/non-specific differences. Frequency domain transformation was applied by computing the Fast Fourier Transform (FFT) spectra from blocks of 512 samples, which resulted in a frequency resolution of 1.953 Hz. Hanning’s window function was applied to overcome spectral leakage phenomena. For compa rison of power at different frequency bands, the areas under the computed power density spectrum in specified frequency ranges, i.e., theta (4–8 Hz), alpha (8–12 Hz), beta (12–30 Hz), and gamma (30–100 Hz) were calculated and averaged. Further, power-spectra were normalized and expressed as percent of total power.

Functional relationships between the BNST/IC LFP and frontal cortical EEG were estimated by means of coherence using the methods described by Halliday et al. (1995). Coherence is one mathematical method of signal processing that can be used to determine the strength of oscillatory synchronizations across the brain networks in different neurological and neuropsychiatric disorders (Bowyer, 2016). Coherence of oscillatory signals provides a frequency-domain measure of the linear phase and amplitude relationships between signals (Alam et al., 2017). In this finite measure of values from 0 to 1, 0 indicates no linear association and 1 indicates a perfect linear association. Coherence is defined as the normalized cross-spectrum according to the formula “Coh x, y (f) = Sxy(f) divided by squared root of Sx(f) -Sy(f),” where x(t) and y(t) are two random, zero-mean processes and Sx(f), Sy(f), and Sxy(f) are the values of their auto-and cross-spectra at a given frequency (f). Representative epochs of 100 s without major artifacts were used for the signal processing. A finite impulse response (FIR) 50 Hz notch filter and 100 Hz low-pass filter was used. Fourier transformation with blocks of 512 samples using a Welch periodogram in a custom MATLAB (MathWorks, Inc.) resulted in a frequency resolution of 1.953 Hz. Hanning’s window function was applied to overcome spectral leakage phenomenon. For comparison of power at different frequency bands, the power of the density spectrum in specified frequency ranges was calculated and the coherence was averaged (Kim et al., 2016).

### Statistics

The statistical procedure of a paired *t*-test was used to verify the difference of spectral power between pre-therapy and post-therapy in the subject. *P*-value < 0.05 was considered as statistically significant.

## Results

### Obsessive-Compulsive Disorder Scale

The MCT sessions resulted in immediate symptomatic changes of the OCD-S items which were scored. Table 2 shows the results of repeated OCD-S measurement during MCT. Only those items are presented that show the main changes.

**Table 2:** Course of selected items of the Obsessive-Compulsive Disorder Scale (OCD-S) ratings: T0 represents the baseline intensity of OCD symptoms before the first MCT session.

Item	T0	T1	T2	T3
How distressing and disabling have your obsessional thoughts/ urges been?	7	4	3	3
How often have you done the following in order to cope with your obsessions?				
- Repeatedly checked	7	4	3	2
- Acted cautiously	8	3	2	2
- Asked for reassurance	7	0	0	0
- Repeated my actions	7	0	0	0
How often have you avoided the following?				
- Social situations	8	1	0	1
- Uncertainty	8	0	1	1
How much do you believe each of the listed beliefs?				
- Obsessional thoughts could change me as a person.	100	80	60	40
- If I think something is contaminated it probably is contaminated	100	60	0	0
- I cannot have peace of mind unless I perform my rituals.	40	40	0	0
- My anxiety will persist if I don’t perform my rituals.	80	70	40	20
- Obsessional thoughts increase the chance of negative events in the future.	40	0	0	0
- Neutralizing my thoughts keeps others/ me safe.	70	20	0	0

*Further measurements show the results after the first MCT session (T1), after the third MCT session (T2), and after the fifth/ last MCT session (T3).*

### Electrophysiological Measures

Several measures in LFP and EEG recordings and their coherence give cues to possible impacts of MCT. In the following the main findings are demonstrated. All results are shown as mean ± standard error of the mean.

**FIGURE 2:**
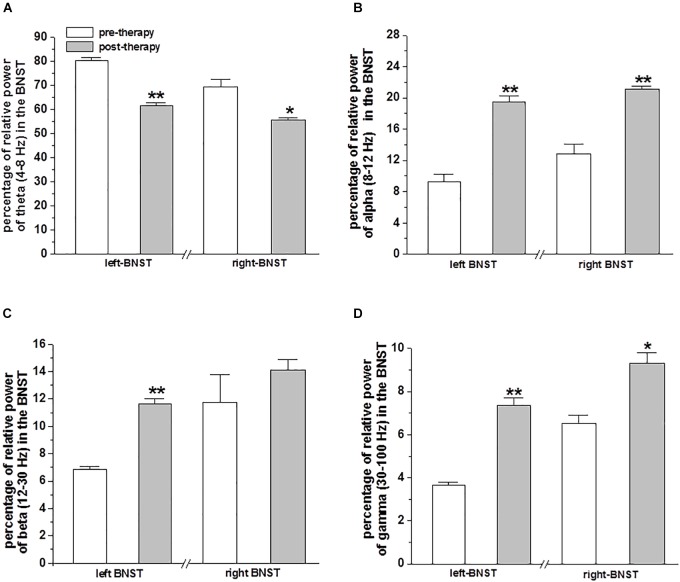
Relative power of LFPs in the BNST from DBS electrodes: The bar plots represent the mean ± SEM percentage of relative power of theta (4–8 Hz) band **(A)**, alpha (8–12 Hz) band **(B)**, beta (12–30 Hz) band **(C)** and gamma (30–100 Hz) band **(D)** oscillatory activity of LFPs recorded in the BNST of left and right electrodes with bipolar reference from contacts 0 vs. 1. Statistical significant differences were determined by paired sample *t*-test and is shown by asterisks (^∗^*p* < 0.05 and ^∗∗^*p* < 0.01).

Prior to MCT therapy, the mean percentage of relative power of theta (4–8 Hz) band LFPs was higher on the left (80.26 ± 1.39%) and on the right (69.19 ± 3.27%) BNST/IC. Whereas, after MCT the relative power of theta band LFPs decreased on the left (61.55 ± 1.04; *p* < 0.01) and right (55.51 ± 1.12%; *p* < 0.04) BNST/IC region, respectively (Figure 2A).

Prior to MCT, the mean percentage of relative power of alpha (8–12 Hz) band LFPs was lower in the left (9.23 ± 1.02%) and on the right (12.81 ± 1.22%) BNST/IC. Whereas, after MCT the relative power of alpha band LFPs increased on the left (19.45 ± 0.77%; *p* < 0.001) and right BNST/IC region (21.07 ± 0.41%; *p* < 0.01; Figure 2B).

Prior to MCT, the mean percentage of relative power of beta (12–30 Hz) band LFPs on the left BNST/IC was lower (6.85 ± 0.22%), whereas, after MCT the relative power of beta LFPs increased in the left BNST/IC region (11.62 ± 0.42%; *p* < 0.01; Figure 2C).

Prior to MCT the mean percentage of relative power of gamma (30–100 Hz) band LFPs was lower on the left (3.64 ± 0.14%) and on the right (6.51 ± 0.38%) BNST/IC. After MCT the relative power of gamma LFPs increased on the left (7.37 ± 0.33%; *p* < 0.01) and right BNST/IC region (9.3 ± 0.5%; *p* < 0.01 and *p* < 0.05; Figure 2D).

**FIGURE 3:**
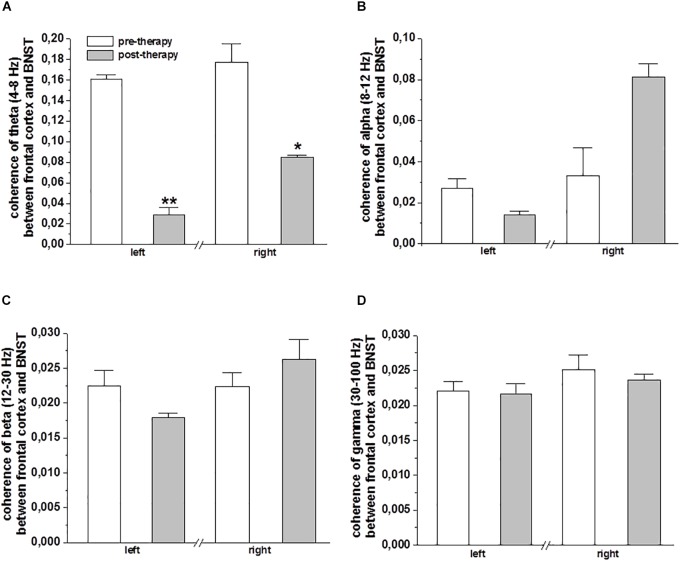
Coherence of frontal cortical EEG and BNST-LFPs of DBS electrodes: The bar plots show the mean ± SEM ratio-transformed coherence of the theta **(A)**, alpha **(B)**, beta **(C)**, and gamma **(D)** frequency band coherence between BNST-LFPs (left and right electrodes, bipolar reference from contacts 0 vs. 1) and the frontal cortical area (EEG left and right frontal cortex). Statistical significance was determined using paired sample *t*-test and is shown by asterisk (^∗^*p* < 0.05 and ^∗∗^*p* < 0.01).

The coherence of oscillatory activity in the frontal cortex and the BNST/IC LFP was analyzed before and after MCT to delineate differences in spectral peak amplitudes and phase locking strength of neuronal network synchronization. A decrease in the mean value of theta frequency band coherence was observed on the left (*p* < 0.001) and right (*p* < 0.05) frontal cortical EEG and BNST/IC LFP after MCT (Figure 3A). No differences in alpha, beta and gamma coherency for the factor therapy were noted (Figure 3B–D).

## Discussion

The present study describes symptom reduction after MCT in trOCD and a possible link between psychotherapeutic interventions and changes in neuronal activity of associated brain network.

Remarkably, OCD symptoms were reduced after only 5 sessions of MCT. Some symptoms remitted already after session 1. Fisher and Wells (Fisher and Wells, 2008) have shown previously that MCT might even be superior to CBT as it appears to be relatively time efficient and an easily delivered treatment. Further, MCT is a straightforward treatment that can be applied even in a laboratory setting and it may be particularly suited to investigate network activity via implanted DBS electrodes.

In our experimental setting, direct recordings from the DBS electrodes revealed a decrease of theta activity and an increase of alpha, beta and gamma-band oscillatory activity in the BNST/IC after MCT. Moreover, MCT was associated with suppression of theta band coherence of the frontal cortex and the BNST/IC.

Our results on basal activity are in line with previous studies who found relatively low alpha and beta power in OCD patients recorded via DBS electrodes in different targets (Guehl et al., 2008; Neumann et al., 2014). More remarkably, clinical and experimental studies have also shown that enhanced cortico-limbic network synchronization in the theta band is correlated with severity of symptoms in OCD, and reduction in such coupling strength may be correlated with clinical improvement (Cavanagh et al., 2011; Voon et al., 2017; Rappel et al., 2018). Enhanced neuronal synchronization in specific frequency bands has been linked to clinical symptoms in movement disorders and disturbed behavior, specifically in theta and beta bands (Nini et al., 1995; Linkenkaer-Hansen et al., 2004; Wilson et al., 2004; Womelsdorf and Fries, 2007). Excessive synchronization therefore is considered pathological with secondary maladaptive signaling (Popovych and Tass, 2014). A recent study has shown an increase in theta activity in the frontal cortex of OCD (Kamaradova et al., 2018). Further, error-related negativity in OCD is thought to be associated with excessive theta synchronization (Luu et al., 2004; Trujillo and Allen, 2007).

Enhanced theta band synchronization, however, may not be specifically attributed to OCD because such a spectrum of synchronization has also been described in patients with dystonia, Tourette syndrome, and psychiatric disorders such as schizophrenia and attention deficit/hyperactivity disorder (Maling et al., 2012; Alam et al., 2015; Kim et al., 2016; Kohl et al., 2016; Neumann et al., 2017, 2018; Won et al., 2018).

This novel paradigm potentially shows much promise to be considered as a possible methodology in future treatment process studies and its main limitation is that the experimental setup was conducted in only one patient, thus far. Also, we cannot fully rule out that the surgical procedure itself had an influence on the initial oscillatory activity in the BNST/IC network, although we started recording of LFP activity only 24 h after electrode implantation to reduce the risk of artifacts, and to give time to the neuronal network to adapt. In our study design it was difficult to rule out the effect of DBS electrodes implantation induced changes to the neuronal activity. However, with regards to current knowledge of DBS in OCD it can be emphasized that the improvement of OCD symptoms most likely only appears after delivery of high frequency electric stimulation. Clinical studies of treatment refractory OCD patients have shown that post-surgery DBS electrodes implantation without current delivery i.e., sham stimulation did not show significant improvement. However, following 12 months of chronic DBS, 4 of 6 patients responded with a decrease of ≥35% in the YBOCS score from baseline (Goodman et al., 2010). Further, studies of DBS in OCD patients have shown altered LFPs before DBS and compensation of abnormal LFP after DBS (Neumann et al., 2014; Pearson et al., 2017). In contrast to DBS therapy our results have shown compensation of altered oscillatory activity of LFPs after MCT in the OCD patient.

No information can be given addressing the question whether symptom reduction through MCT alone in this treatment resistant case would have lasted as DBS started once the IPG was implanted. The patient initially came to receive DBS. He additionally participated in the described paradigm, but was then treated and monitored according to DBS protocol.

## Conclusion

We here present an experimental paradigm to directly investigate neuronal oscillatory activity in patients with trOCD before and after application of MCT by recording LFP via implanted DBS electrodes. Our results suggest that a dominant decrease in the theta frequency band in the BNST/IC and in frontal cortical coherency, and an increase in the relative power of alpha, beta and broad band gamma frequency oscillatory activity in the BNST/IC may be associated with OCD symptom reduction by MCT. Our preliminary results may give possible cues for neuronal circuitry changes in OCD secondary to psychotherapy.

## Ethics Statement

This study was carried out in accordance with the recommendations of the ethic committee of the Hannover Medical School. The subject received oral and written information about the study, participation was voluntary and he gave written informed consent in accordance with the Declaration of Helsinki. The protocol was approved by the ethic committee of the Hannover Medical School.

## Author Contributions

LW, MA, HH, KS, JK, and KK planned the original concept. JK and AS performed surgery. MA and HH were in charge of data recordings and handling of all equipment needed. LW performed the intervention sessions. MA, DM, XJ, IH, and KS did the data analysis. All authors contributed to writing the paper and interpretation of the results.

## Conflict of Interest Statement

The authors declare that the research was conducted in the absence of any commercial or financial relationships that could be construed as a potential conflict of interest.
